# Retrospective Analysis of Dental Implant Stability in Relation to Mandibular Bone Density and Cortical Thickness in Osteopenic and Osteoporotic Patients

**DOI:** 10.3390/jcm14041339

**Published:** 2025-02-18

**Authors:** Monika Probst, Florian Probst, Matthias Tröltzsch, Matthias Bruckbauer, Alexander W. Marka, Gustav Andreisek, Thomas Frauenfelder, Egon Burian

**Affiliations:** 1 Department of Diagnostic and Interventional Neuroradiology, Klinikum Rechts der Isar, School of Medicine, Technical University of Munich, 80636 Munich, Germany; monika.probst@tum.de; 2 Department of Oral and Maxillofacial Surgery and Facial Plastic Surgery, University of Munich, 80636 Munich, Germany; flo.probst@web.de (F.P.); matthias.troeltzsch@med.uni-muenchen.de (M.T.); 3 Department of Oral and Maxillofacial Surgery, University of Salzburg, 5020 Salzburg, Austria; matthias.bruckbauer@gmx.de; 4 Department of Diagnostic and Interventional Radiology, Klinikum Rechts der Isar, School of Medicine, Technical University of Munich, 80636 Munich, Germany; alexander.marka@tum.de; 5 Department of Diagnostic and Interventional Radiology, Cantonal Hospital Frauenfeld, 8500 Frauenfeld, Switzerland; gustav.andreisek@team-radiologie.ch; 6 Faculty of Medicine, University of Zurich, 8057 Zurich, Switzerland; thomas.frauenfelder@usz.ch; 7 Institute of Diagnostic and Interventional Radiology, University Hospital Zurich, 8057 Zurich, Switzerland

**Keywords:** osteoporosis, mandibular bone density, dental implants, dual-energy X-ray absorptiometry, cone beam computed tomography, bone mineral density

## Abstract

**Background/Objectives:** The aim of this study is to examine the relationship between cervical spine and jaw bone mineral density (BMD) and assess how cortical thickness and BMD influence primary implant stability (PIS) across different bone health conditions. **Methods:** The study included 29 patients (mean age: 63.7 ± 13.7 years; 13 women) and 15 healthy controls (mean age: 25.3 ± 3.0 years; seven women). Cervical spine (C2–C4) and mandibular BMD were evaluated using asynchronous calibration and manual segmentation. Cortical thickness was measured, and primary implant stability was assessed via insertion torque in Newton centimeters (Ncm). **Results:** Cervical spine BMD was significantly lower in the patient group compared to controls (203.0 ± 51.0 vs. 252.0 ± 21.7 mg/dL, *p* < 0.0001). No significant correlation was found between mandibular BMD and cervical spine BMD in both groups (patients: ρ = 0.1287; *p* = 0.506, controls r = −0.1214; *p* = 0.667). Linear regression analysis revealed that cortical thickness alone, not implant site BMD, significantly influenced PIS (F(2.74) = 5.597, *p* = 0.005). **Conclusions:** Asynchronous calibration accurately quantifies cervical and mandibular BMD. Cortical thickness rather than overall bone density emerges as a critical factor in determining implant stability. These findings suggest that clinicians should prioritize cortical thickness assessment when planning dental implant procedures, potentially improving outcomes across diverse patient bone health profiles.

## 1. Introduction

Osteoporosis, characterized by decreased bone mineral density (BMD) and altered bone microarchitecture, presents a significant challenge in dental implantology. While the impact of osteoporosis on the skeletal system is well documented, its effects on mandibular bone quality and dental implant stability remain controversial [1].

There is a plentitude of the literature on the heterogeneity of BMD decrease within the human skeleton, with an earlier and more severe deterioration of trabecular bone microarchitecture compared to cortical bone [2,3,4]. Previous studies have tried to bring light to possible correlations between BMD calculated by dual-energy X-ray absorptiometry (DXA) and qualitative bone quality assessed by means of dental radiography (OPT) and cone beam computed tomography (CBCT) [5,6,7]. In a systematic review conducted by Calciolari et al. comparing BMD in osteoporotic patients with a microarchitecture in the alveolar ridge, no clear correlation between the two could be established [7]. Until today there is a certain ambiguity regarding the correlation between BMD loss within osteoporosis and implant failure, between BMD decrease and the microarchitectural decay of trabecular bone in the jaw, and also between the structural composition of the implant site and survival rate of inserted implants [8,9,10].

This discrepancy highlights a critical gap in our understanding of how systemic bone loss translates to local mandibular conditions. Stratifying the risk for implant failure in patients with decreased BMD is of great importance from a medical and economic point of view [11,12,13]. While a preoperative assessment of bone quality typically relies on OPT or cone beam computed tomography (CBCT), research suggests these methods may lack precision in accurately determining bone quality [14,15,16,17,18]. The relationship between the mandibular bone structure and primary PIS also remains unclear. Despite numerous studies, there is no consensus on how BMD loss in osteoporosis affects implant failure rates or how the structural composition of the implant site influences implant survival [8,19,20].

To address these knowledge gaps, this exploratory, retrospective, and cross-sectional study aims to analyze the correlation between cervical spine BMD and mandibular bone density. Furthermore, our aim was to assess which structural factors of the mandible influence primary implant stability. By investigating the relationship between systemic osteoporosis and mandibular bone quality, we aim to improve risk assessment and treatment planning for dental implant patients with reduced bone mineral density.

## 2. Materials and Methods

### 2.1. Study Design

For this study, patients were retrospectively included. The present study was carried out between November 2018 and December 2020 at the Department of Diagnostic and Interventional Neuroradiology, Klinikum rechts der Isar, Technical University of Munich, Germany. Implant patients were evaluated based on their BMD values, which are determined in the spine by CT imaging, according to the reference values of Kaesmacher et al., divided into three groups [21]. The three groups of “osteoporosis”, osteopenia”, and “normal” are compared based on their BMD values in the lower jaw and based on the insertion torque in Ncm of the implants.

A comparison group of young and healthy people was formed. These subjects served as a reference group of healthy subjects to be compared with the patient group based on their BMD values in the cervical spine and in the mandible.

The study received approval from the relevant institutional review board (Technical University of Munich: ref. no. 413/17 S).

### 2.2. Patients and Control Group

The inclusion criteria were as follows:

Patients received a CT scan in order to receive computer-guided implant planning. During the procedure, the insertion torque, which was necessary to achieve primary stability, was documented in daily routine by using a torque measuring device (Newton cm (Ncm)).

Patients were excluded if they had a known underlying neoplastic disease and/or had a history of radiation therapy to the oral and maxillofacial region or if they received intravenous oral antiresorptive treatment.

From the initially included 49 patients, 20 had to be excluded due to the described criteria. The reasons for the subsequent exclusion were either that no vertebral bodies in the cervical spine were visible in the CT scan or that no implant was placed in the mandible.

The control group consisted of 15 subjects who underwent CT imaging of the facial skull and cervical spine at the university hospital Klinikum rechts der Isar, Technical University of Munich, due to trauma. This cohort received the CT scan due to a history of trauma. The young age of these trauma patients ensured that the possibility of suffering from osteoporosis was low. As the phantom for calculating the Hounsfield units to BMD was performed asynchronously, meaning that the conversion factor can be calculated for each scanner, a comparison of two different groups scanned on two different scanners was possible.

The inclusion criteria for the control group were as follows: patients who underwent CT of the skull between 20 and 30. Exclusion criteria were if severe metal artifacts or fractures in the area of the jaw and/or cervical spine could be seen.

### 2.3. Computed Tomography (CT)

The reference body was scanned with a slice thickness of 0.8 mm. The spiral pitch factor was 0.55. The tube voltage was 120 kV, and the tube current was 88 mAs. The image matrix had 512 × 512 pixels, and the FOV (“field of view”) was 195 mm in size. In each scan, the Hounsfield units (HUs) of the corresponding cervical vertebral bodies and the mandible were extracted.

### 2.4. Image Analysis and Segmentation

The CT datasets were acquired for preoperative planning in the course of implantation. Each CT (Phillips Brilliance 16 CT scanner, Philips Medical Systems, Amsterdam, The Netherlands) was conducted by a radiology practice (Practice for Radiology located in Ansbach Hospital, Ansbach, Germany). All implantations (3.1 mm, 4.1 mm, and 4.8 mm diameter and 10 mm length, titanium implants, Straumann, Institute Straumann AG, Basel, Switzerland) were performed by experienced specialists for oral and maxillofacial surgery (Practice for Oral and Maxillofacial Surgery Dr. Dr. Tröltzsch, Center for Dentistry, Oral and Maxillofacial Medicine, Ansbach, Germany). The data were collected and archived between 2009 and 2017. The CT data were transferred in anonymized form in DICOM format to the research PACS (PACS, Sectra IDS7, Sectra AB, Linköping, Sweden) of the Institute of Radiology or the Department of Diagnostic and Interventional Neuroradiology.

For the analysis of the attenuation values (HUs), sagittal reformations with the 10 mm slice thickness and a 1 mm space between each slice was reformatted. Subsequently, circular ROIs were manually placed in the ventral halves of the trabecular compartment of the cervical vertebral bodies C2 to C4 in the sagittal plane using the attenuation measurement tool, thus determining the attenuation values in HU. The ROI dimensions were standardized to half the superior–inferior extent of the vertebral body. The attenuation values were measured using the attenuation measurement tool provided by PACS.

The mean values of attenuation in HUs within the ROI were converted into volumetric BMD values using a conversion equation obtained through asynchronous phantom calibration.

**Figure 1:**
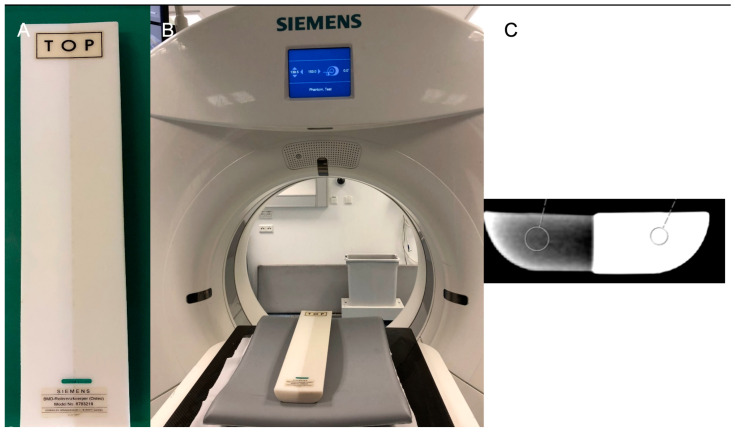
(**A**) The 2-element calibration phantom. (**B**) The CT with the calibration phantom. In (**C**), the images are shown after the CT scan.

This measurement method has proven to be reliable in several studies [21,22]. For this purpose, the CT scanner at the radiology practice in Ansbach was calibrated using a two-element calibration phantom (Osteo Phantom, Siemens Healthcare AG, Erlangen, Germany), which consists of a water-like and a bone-like phase. This phantom is a semicircular cylinder with two separate chambers, the water equivalent containing 91.3% PE, 5.5% CaCO_3_ and 3.2% MgO (Figure 1).

### 2.5. Asynchronous Calibration and Establishing a Conversion Equation for Retrospective BMD Calculation

Asynchronous calibration means that no calibration phantom is required during the actual CT scan. Instead, asynchronous CT uses data obtained independently from other scans to calculate the quantitative correlation of HUs and BMD. Thus, HUs can be transferred directly to BMD values. This approach has been shown in multiple studies previously [21,22,23].

Using linear regression models, HU-to-BMD conversion equations were established based on defined phantom values in two CT scanners (Ansbach Radiology and university hospital Klinikum rechts der Isar, Technical University of Munich). This made it possible to convert the Hounsfield values measured in the CT into valid BMD values with the unit mg/mL.

**Figure 2:**
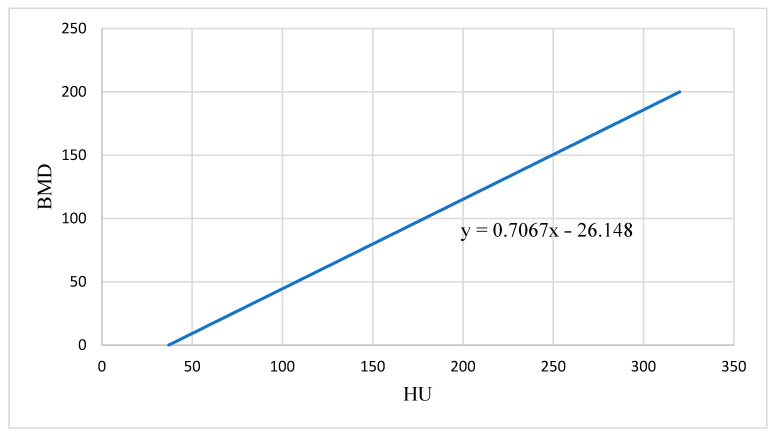
Conversion equation with best correlation of Hounsfield units (HUs) and bone mineral density (BMD).

The conversion equation of the Philips Brilliance 16 CT scanner from our cooperative practice was y = 0.7067x − 26.148 (Figure 2).

The conversion equation of the CT scanner Siemens Somatom Definition AS+ of the hospital on the right of the Isar was y = 0.6897x − 34.483.

After converting the HUs into BMD values, the patients were divided into the subgroups “osteoporosis”, “osteopenia”, and “normal”.

### 2.6. Segmentation

**Figure 3:**
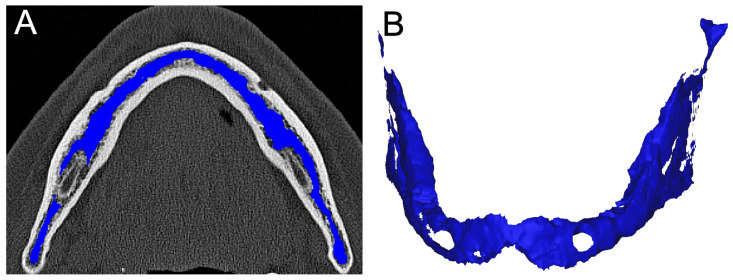
Segmentation example for mandibular BMD calculation. (**A**) An axial reformation of the CT dataset and (**B**) a 3D reconstruction with bone marrow colored blue. This figure visualizes which anatomic regions form the mandibular BMD.

**Figure 4:**
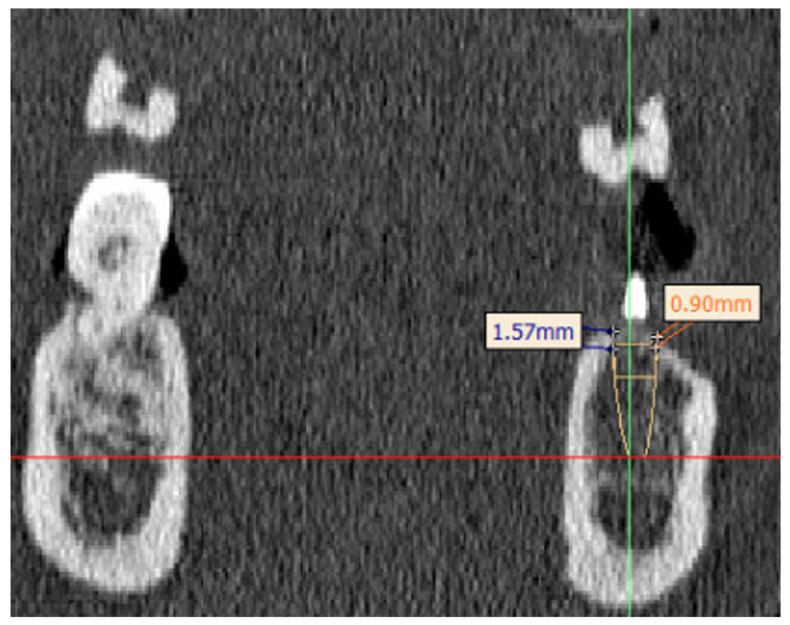
Planning dataset before implant placement. This figure showcases the ROI placement with regard to implant site planning. In yellow the implant diameter is shown, in orange the cortical thickness is measured on the buccal side, in blue the cortical thickness is measured at the lingual side. In red the horizontal line is depicted, in light green the vertical line is shown.

For implant planning, each patient received preoperative CT imaging of the viscerocranium, which also imaged the cranial cervical spine from C2 to C4. The data were saved in DICOM format and loaded into the segmentation program Mimics 22.0 (Materialise NV, Leuven, Belgium). First, the scans were segmented in the region of the implants; secondly, the entire cancellous bone of the lower jaw was segmented (Figure 3 and Figure 4). This made it possible to determine the BMD values of the two regions mentioned.

Using the planning template located in the patient’s oral cavity, the actual position of the implant was reconstructed, and an imp lant analog cylinder (ROIs) was placed at this location. The position was determined by senior oral and maxillofacial surgeons with dental implantology specialization.

The HU values were measured in the ROI and then converted into BMD values. The following areas were included in the ROI:

(1) The implant volume of the cylinder;
(2) The surroundings of the dental implant area (approximately 0.66 mm);
(3) The cancellous bone at the insertion site.

At the same location, the vestibular and lingual cortical thickness at the level of the implant shoulder was measured.

The cancellous bone was segmented using the following method:

First, a mask with a visually determined threshold was created. A threshold (lower and upper HU limits) was chosen so that the entire cancellous bone is segmented within the mandible. A distance of at least 0.5 mm to the adjacent teeth ensured that no other structures like teeth falsify the values. The nerve canal of the inferior alveolar nerve was excluded on both sides.

### 2.7. Statistical Analysis

Statistical analysis was performed using IBM SPSS Statistics for Windows, Version 27.0 (IBM Corp., Armonk, NY, USA). To convert HUs to BMD values, a phantomless HU-to-BMD conversion equation was used. The normal distribution of the data was checked using the Kolmogorov–Smirnov test. The significance level α was set at 0.05, with *p*-values < 0.05 considered significant.

For correlation analyses, Pearson’s correlation was used for normally distributed data, while Spearman’s correlation was applied for non-normally distributed data. This choice ensures appropriate analysis based on data distribution. To compare BMD in the cervical spine between the test group and patient group, the independent samples t-test with Welch’s correction was employed due to unequal sample sizes. This test is robust against violations of homogeneity of variance.

The Mann–Whitney U test was used to compare BMD in the lower jaw between test subjects and patients for non-normally distributed data, as it does not assume normality. A one-way ANOVA was conducted to compare BMD in the lower jaw among the “osteoporosis”, “osteopenia”, and “normal” groups. This test was chosen, as it allows for a comparison of means across multiple groups.

A linear correlation analysis using Spearman’s rho coefficient was performed to determine relationships between various morphologic factors on the primary implant stability (PIS) of the implants, suitable for ordinal or non-normally distributed data.

The Kruskal–Wallis test (H test) was used to compare primary stability (in Ncm) among the three patient groups, as it is appropriate for comparing three or more groups with non-normally distributed data.

For significant pairwise comparisons, the effect size r was calculated using the following formula: r = |z/√N|, where z is the standard test statistic and N is the sample size. This provides a standardized measure of the strength of the observed effects.

## 3. Results

### 3.1. Descriptive Statistics

From the initially recorded 49 patients, 20 had to be excluded due to the criteria described. Finally, the patient group consisted of 29 patients (age: 63.7 ± 13.7 years, age range: 31–88, 13 women). The control group consisted of 15 subjects (age: 25.3 ± 3.0 years, age range: 20–30, 7 women).

### 3.2. BMD Comparison Between Patients and Controls

**Figure 5:**
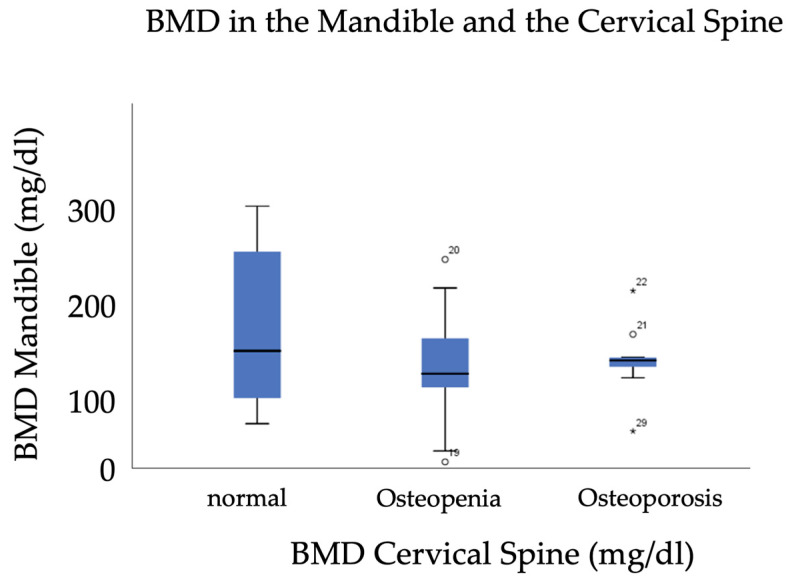
Distribution of mandibular BMD in patients with normal, osteopenic, and osteoporotic BMD in the cervical spine. Circles and * represent outliers from the median.

The independent samples *t*-test showed a statistically significant difference in the BMD of the cervical spine between the patient group (203.0 ± 51.0 mg/dL ± SD) and the subject group (252.0 mg/dL ± 21.7, *p* < 0.0001). There was no statistically significant difference in mandibular bone density between the patient (129.3 mg/dL ± 74.6 SD) and control group (119.4 mg/dL ± 49.6, *p* = 0.473) (Figure 5).

### 3.3. Correlation of Vertebral and Mandibular BMD and Age

The BMD in the cervical spine of the patient group showed a significant correlation with age (ρ = −0.6402; *p* = 0.0002, CI = −0.8191 to −0.3476). Spearman’s rho correlation analysis did not reveal a statistically significant relationship between age and BMD of the mandible (ρ = 0.09780; *p* = 0.6137, CI = 0.2891 to 0.4573).

### 3.4. Correlation and Regression Analyses of Cortical Thickness, Insertion Torque, and Primary Implant Stability

The Spearman rho correlation analysis shows a statistically significant positive relationship between the cortical thickness, the insertion torque, and the PIS (ρ = 0.288; *p* = 0.011, CI = 0.06121 to 0.4857). A linear regression analysis shows that the cortical thickness but not the BMD of the implant site has an influence on the PIS of the implant in Ncm (F(2.74) = 5.597, *p* = 0.005).

### 3.5. Subgroup Analysis of Different Vertebral BMD Levels

Furthermore, a subgroup analysis was conducted to stratify the impact of a gradual decreasing vertebral BMD in patients suffering from osteopenia and osteoporosis. Patients with the osteoporotic (n = 9), osteopenic (n = 12), and normal BMD (n = 8) conditions in the cervical spine showed no significant differences in regard to mandibular BMD according to the ANOVA (*p* = 0.395). The Kruskal–Wallis test showed that there is a significant difference between the “normal” and “osteoporosis” groups (z = −2.399, *p* = 0.049) and between the “normal” and “osteopenia” groups in terms of the PIS in Ncm (z = −2.931, *p* = 0.010). Effect sizes were r = 0.45 and r = 0.33 and can be classified as moderate.

## 4. Discussion

This study investigated the relationship between various osseous characteristics and primary implant stability (PIS) in dental implantation. Our findings reveal important insights into the factors influencing PIS and challenge some existing assumptions about bone quality assessment in implant dentistry. The impact of the asynchronously calculated BMD of the cervical spine, the mandible, and measured cortical thickness was correlated with insertion torques during the implantation procedure. Löffler et al. described the validity of asynchronous calibration in the context of extracting BMD values retrospectively using a scan phantom [24]. The first finding of our work was that the cervical spine and the mandible have distinct BMD and structural hard tissue composition. The BMD of the cervical spine falling in the osteoporotic range did not correlate with reduced mandibular BMD. Furthermore, PIS in osteoporotic patients was not significantly reduced compared to bone healthy patients. The most important factor associated with PIS was cortical thickness.

In clinical practice, it is of critical importance to have reliable measures for PIS, which can be expected in the course of dental implantation. There is a plentitude of the literature on different structural requirements associated with implant survival rate like cortical thickness, general BMD, and bone composition at the implantation site [25,26]. Different imaging modalities have been described for an accurate assessment of the implantation site and presurgical planning, from dental radiographs to panoramic radiographs and cross-sectional imaging like CT, CBCT, and MRI [21,22,23]. For presurgical purposes, CBCT allows for HU conversion and an indirect quantitative assessment of the alveolar ridge [27]. Although nowadays, CBCT is the dominating imaging technique in daily practice for three-dimensional implantation site visualization; using CT in the course of the presented study setup allowed for generating quantitative insight into BMD and implant interplay.

Considering different imaging techniques for bone evaluation, there is DXA and CBCT. CBCT has the advantage of high spatial resolution compared to CT lower radiation doses [28]. However, CBCT does not allow for BMD calculation, as only arbitrary units can be extracted. The gold standard of BMD evaluation is DXA [29]. But DXA is only applicable in settings with specific medical questions like bone status and is not suitable for the high-resolution imaging of bone [24].

In the next paragraphs, we further delve into the different aspects of PIS with regard to our presented results. First, we discuss the primary finding, namely that PIS has a primary correlation with cortical thickness.

Our results demonstrate a strong positive correlation between cortical thickness and insertion torque, indicating that cortical thickness is the most significant factor influencing PIS. A total of 15 years ago Roze et al. showed the importance of cortical thickness on PIS [30]. The importance of cortical bone in PIS can be attributed to its role in the initial mechanical stability of the implant and the transmission of occlusal loading forces to the peri-implant bone tissue.

The clinical implication of this finding is significant. A special focus should be put on the assessment of cortical thickness in pre-operative planning. Surgical techniques that maximize engagement with the cortical bone may lead to improved primary stability and potentially better long-term outcomes. There are also other more recent studies which are consistent with the present study [31,32]. In turn, according to Merheb et al., both the cortical thickness and the local bone density have a significant influence on the PIS [32].

Interestingly, our study found no significant correlation between BMD in the cervical spine and the mandible. This lack of correlation suggests that these anatomical regions may have distinct mechanisms of bone aging and deterioration. This finding challenges the assumption that systemic BMD measurements, such as those from the spine or hip, can reliably predict local bone quality in the jaw.

The clinical implication is that systemic BMD measurements, often used to diagnose conditions like osteoporosis, may not be reliable indicators of mandibular bone quality for dental implant planning. This underscores the need for a site-specific assessment of bone quality in the jaw. Systemic BMD measurements may have limited utility in predicting mandibular bone quality for implant planning.

Furthermore, the presented results reveal no significant difference in PIS between osteoporotic and non-osteoporotic patients. This finding is consistent with high-level evidence from systematic reviews and meta-analyses by Radi et al. and Schimmel et al., which reported similar 5-year implant survival rates in osteoporotic and healthy patients [19,33].

This result challenges the notion that osteoporosis is a contraindication for dental implant therapy. It suggests that local factors, particularly cortical thickness, may be more critical for implant stability than systemic bone health. However, it is important to note that while initial stability may not be significantly affected, long-term outcomes and the potential need for modified healing protocols in osteoporotic patients warrant further investigation.

Given the amount of data on this complex interplay of bone pathophysiology and dental implants, there are studies which support our results on the impact of BMD on PIS [11,34,35]. However, there is also research that supports a more central role for BMD in the context of dental implant planning [32,36]. These controversial results highlight the complexity of this research topic. In clinical practice, a personalized approach taking into account patients’ systemic and local bone status into account and deciding which rehabilitative approach is the most suitable in each individual patient.

Several limitations of this study should be acknowledged. The retrospective design introduces potential selection bias. Image quality was compromised by metal artifacts in certain cases, which could affect HU measurement accuracy. The manual documentation of insertion torque in 5 Ncm increments may lack precision. The relatively small and heterogeneous study cohort limits generalizability.

To address these limitations and further advance our understanding, there are several steps that can be taken. First of all, prospective studies with larger, more homogeneous cohorts are needed. The utilization of artifact-reduced CT sequences which minimize beam hardening artifacts are recommended. Furthermore, using more precise methods for measuring PIS, such as resonance frequency analysis, could be integrated in the study protocol. Lastly, investigating the long-term outcomes of implants in patients with varying cortical thicknesses and BMD values would offer valuable insights into the clinical implications of these findings over time. These research directions would collectively address the current study’s limitations and contribute to a more comprehensive understanding of the factors influencing implant stability and success.

This study highlights the critical role of cortical thickness in determining PIS, surpassing the influence of local or systemic BMD measurements. The lack of correlation between mandibular and vertebral BMD challenges current assumptions about bone quality assessment in implant dentistry. While osteoporosis does not appear to significantly impact initial implant stability, the site-specific evaluation of bone parameters, particularly cortical thickness, is crucial for optimal treatment planning.

## 5. Conclusions

The presented results show that cortical thickness might be a more important factor for PIS than mandibular BMD. This finding underscores the importance of a site-specific assessment of cortical thickness in pre-operative planning for dental implants.

## Data Availability

The original contributions presented in this study are included in the article. Further inquiries can be directed to the corresponding author.

## Abbreviations

The following abbreviations are used in this manuscript:

BMD	Bone mineral density
CBCT	Cone beam computed tomography
CT	Computed tomography
DXA	Dual-energy X-ray absorptiometry
ICC	Intraclass correlation coefficient
MD	Mean difference
MRI	Magnetic resonance imaging
OPT	Conventional panoramic radiography
PACS	Picture archiving and communication system
PIS	Primary implant stability
